# Imaging features and clinical evaluation of pulmonary nodules in children

**DOI:** 10.3389/fonc.2024.1385600

**Published:** 2024-08-08

**Authors:** Muheremu Dilimulati, Shuhua Yuan, Hejun Jiang, Yahua Wang, Hui Ma, Shiyu Shen, Jilei Lin, Jiande Chen, Yong Yin

**Affiliations:** ^1^ Department of Respiratory Medicine, Shanghai Children’s Medical Center, School of Medicine, Shanghai Jiao Tong University, Shanghai, China; ^2^ Child Health Advocacy Institute, China Hospital Development Institute, Shanghai Jiao Tong University, Shanghai, China; ^3^ Department of Respiratory Medicine, Sanya Women and Children’s Hospital Affiliated to Hainan Medical College, Hainan Branch of Shanghai Children’s Medical Center, Shanghai Jiao Tong University School of Medicine, Sanya, Hainan, China; ^4^ Department of Respiratory Medicine, Linyi Maternal and Child Healthcare Hospital, Linyi Branch of Shanghai Children’s Medical Center, Shanghai Jiao Tong University School of Medicine, Linyi, Shandong, China; ^5^ Shanghai Children’s Medical Center Pediatric Medical Complex (Pudong), Shanghai, China; ^6^ Pediatric AI Clinical Application and Research Center, Shanghai Children’s Medical Center, Shanghai, China; ^7^ Shanghai Engineering Research Center of Intelligence Pediatrics (SERCIP), Shanghai, China

**Keywords:** pulmonary nodule, children, benign and malignant differentiation, diagnostic model, computed tomography

## Abstract

**Background:**

With the widespread use of computed tomography (CT), the detection rate of pulmonary nodules in children has gradually increased. Due to the lack of epidemiological evidence and clinical guideline on pulmonary nodule treatment in children, we aimed to provide a reference for the clinical diagnosis and management of pediatirc pulmonary nodules.

**Methods:**

This retrospective study collected consecutive cases from April 2012 to July 2021 in the Shanghai Children’s Medical Center. The sample included children with pulmonary nodules on chest CT scans and met the inclusion criteria. All patients were categorized into tumor and non-tumor groups by pre-CT clinical diagnosis. Nodule characteristics between groups were analyzed. To establish a clinical assessment model for the benign versus malignant pulmonary nodules, patients who have been followed-up for three months were detected and a decision tree model for nodule malignancy prediction was constructed and validated.

**Results:**

The sample comprised 1341 patients with an average age of 7.2 ± 4.6 years. More than half of them (51.7%) were diagnosed with malignancies before CT scan. 48.3% were diagnosed with non-tumor diseases or healthy. Compared to non-tumor group, children with tumor were more likely to have multiple nodules in both lungs, with larger size and often be accompanied by osteolytic or mass lesions. Based on the decision tree model, patients’ history of malignancies, nodules diameter size≥5mm, and specific nodule distribution (multiple in both lungs, multiple in the right lung or solitary in the upper or middle right lobe) were important potential predictors for malignity. In the validation set, sensitivity, specificity and AUC were 0.855, 0.833 and 0.828 (95%CI: 0.712-0.909), respectively.

**Conclusion:**

This study conducted a clinical assessment model to differentiate benignity and malignancy of pediatric pulmonary nodules. We suggested that a nodule’s diameter, distribution and patient’s history of malignancies are predictable factors in benign or malignant determination.

## 1 Introduction

Pulmonary nodules are defined as round, oval or irregular lesions, surrounded by lung parenchyma, with a diameter of ≤3cm (1), and can be ground glass, solid, or contain both solid and ground glass components (2). Pulmonary nodules are more common in adults because primary lung cancer is one of the most common cancers in this population. The etiology of pediatric pulmonary nodules differs from that in adults, and often lack specificity. Common causes in pediatric pulmonary nodules include infectious lung diseases, pulmonary metastases, connective tissue diseases and other less common etiologies (such as chronic granulomatous disease, pulmonary nodulosis and multiple pulmonary arteriovenous fistulas) (3–5).

In recent years, the widespread use of chest computed tomography (CT) has made an increase in the detection of pediatric pulmonary nodules. The prevalence of incidental lung nodules in healthy children is not low. Alves et al. (6) evaluated preoperative thoracic CT scans of 99 children who were scheduled for surgery for pectus carinatum or excavatum and found the detection rate of nodule was 75%. In 2017, Samim and colleagues found a detection rate of pulmonary nodules on CT scan was 38.0% in pediatric patients after trauma (7). Similarly, researchers (8) retrospectively reviewed 259 trauma chest CT scans in pediatric patients and demonstrated a total of 86 patients (33%) with incidental pulmonary nodules. A large proportion of the studies revealed that pulmonary nodules are also not uncommonly identified in the pediatric population with extra thoracic malignancies. Silva and colleagues (9) reviewed 488 patients with a noncentral nervous system solid tumor or lymphoma and identified 111 patients (23%) with pulmonary nodules on the presentation CT scan. Recently, a study (10) reported more than one-fifth had pulmonary nodules in 316 pediatric patients with rhabdomyosarcoma. Another study discovered that 57.0% of the nodules detected by CT were malignant in children with osteosarcoma (11). These existing studies evaluated features of pulmonary nodules in children, but there is still no consensus on reliable features to determine a benign incidental pulmonary nodule versus metastatic disease. Therefore, the pulmonary nodule in pediatric population is not uncommonly encountered and becomes a challenging situation.

There were few accepted criteria for pediatric pulmonary nodule evaluation. In 2014, an American study found that 40% of pediatric pulmonologists referred to the Fleischner guidelines (12). This guideline aims at patients ≥35 years old, indicating a tendency towards overtreatment of pediatric pulmonary nodules (13). The development of standard guidelines for pediatric pulmonary nodules is an urgent demand (14). Although lung biopsy is the gold standard for nodule diagnosis, it is not the first choice due to its invasiveness. Chest CT is the first line guide for determining benignity or malignancy (6, 8, 15, 16). Our study is designed to provide a reference for clinical diagnosis, treatment, and management of pulmonary nodules for children.

## 2 Materials and methods

### 2.1 Study design and data collection

We retrospectively collected consecutive cases from April 2012 to July 2021 in the Shanghai Children’s Medical Center. The inclusion criteria were as follows: (1) age <18 years; (2) first detection of pulmonary nodules on CT; (3) pulmonary nodules with a diameter ≤3cm, round or irregular in shape and presenting as high-density shadows. The exclusion criteria were: (1) pulmonary mass with a diameter >3cm on CT; (2) missing clinical data or radiological data was unavailable. Patients’ demographic information (age, sex), clinical record (clinical department, respiratory symptoms, Pre-CT clinical diagnosis, past medical history and follow-up information etc.) and chest CT data (nodule diameter, density, distribution, accompanied radiological features, and radiological diagnosis) were collected by the electronic medical record system. All subjects were categorized into tumor and non-tumor groups by pre-CT diagnosis. Patients having infections, non-tumor hematological diseases, autoimmune diseases, immunodeficiencies and other diagnosis like trauma were set into non-tumor group.

To establish a clinical assessment model, children with pulmonary nodule follow-up for ≥3 months were subdivided into malignant and benign nodule subgroups. Data collected in subgroups met the following criteria: (1) radiological diagnosis was made by two experienced radiologists’ analysis of changes in the imaging characteristics of the nodules during follow-up (including size, density, distribution, etc.); (2) clinical judgment of the nodule was made by clinician based on the therapeutic effect (including tumor chemotherapy, anti-infection, anti-inflammatory treatment, etc.). The collected samples were then randomly divided into a training set and a validation set, and the features of the training set data were analyzed to build a decision tree algorithm using R language.

The study was approved by the Institutional Review Board of the Shanghai Children’s Medical Center, Shanghai Jiao Tong University (SCMCIRB-K2022085-2).

### 2.2 Statistical analysis

SPSS 26.0 software was utilized for statistical analysis of demographic information, clinical record and chest CT data. The t-test, Mann-Whitney U test and χ2 test were used to analyze data between groups. A *P*-value <0.05 was considered statistically significant.

To fit the potential nonlinear relationships in the data and provide an intuitive explanation of the decision paths, we used a decision tree model to predict the malignancy of nodules. It was constructed using the rpart () and prp () functions from the rpart package in R version 4.2.2. The training dataset was used to grow the decision tree model. Five-fold cross-validation and grid search were employed on the training set to tune the hyperparameters. The model achieved optimal performance when the nodes of supersets and subsets were set to 10 and 5, respectively, and the maximum growth depth was set to 3 layers. The cutoff value was determined from the maximum value of the Youden index, and the corresponding sensitivity and specificity were calculated.

## 3 Results

### 3.1 Clinical characteristics and CT features between tumor and non-tumor groups

A total of 1341 children were detected with pulmonary nodules for the first time on chest CT scan, with 736 males (54.9%) and 605 females (45.1%). The average age was 7.2 ± 4.6 years. In **Table 1**, more than half of patients (n=693, 51.7%) were diagnosed with malignant tumors before the CT examination.

**Table 1:** Demographic and clinical features of children with pulmonary nodules detected by chest CT.

Age, years mean (SD))	7.2 (4.6)
Sex	
Male	736 (54.9%)
Female	605 (45.1%)
Departments	
Respiratory	129 (9.7%)
Hematology & Oncology	622 (46.4%)
Other internal medicine	365 (27.2%)
Surgery	211 (15.7%)
Otolaryngology	12 (0.9%)
Traditional Chinese medicine	2 (0.1%)
Pre-CT diagnosis	
Tumor	693 (51.7%)
Respiratory infections	228 (17.0%)
Infection of other systems	18 (1.3%)
Immunodeficiencies	22 (1.7%)
Autoimmune diseases	75 (5.6%)
Non-tumor hematological diseases	70 (5.2%)
Others	235 (17.5%)
Respiratory symptoms	
Yes	232 (17.3%)
No	1109 (82.7%)
Whether follow-up	
Yes	463 (34.5%)
No	878 (65.5%)

Children with tumor had significantly larger pulmonary nodules and were more likely to have multiple nodules in both lungs, often be accompanied by osteolytic or mass lesions on imaging. In non-tumor group, nodules were frequently located in the lower right lobe and were accompanied by patchy and striated shadows on imaging, children in this group showed more respiratory symptoms. Gender and nodule density showed no difference between groups (**Table 2**).

**Table 2:** Comparison of general data and pulmonary nodule characteristics between children in the tumor and non-tumor groups.

	All	Tumor	Non-tumor	t/Z/x2	*P* value
	N=1341	N=693	N=648		
Age (years)	7.2 (4.6)	6.7 (4.7)	7.7 (4.3)	4.097	<0.001
Sex				0.750	0.386
Male	735 (54.9%)	388 (56.0%)	347 (53.5%)		
Female	606 (45.1%)	305 (44.0%)	301 (46.5%)		
Respiratory symptoms				102.240	<0.001
Yes	232 (17.3%)	50 (7.2%)	182 (28.1%)		
No	1109 (82.7%)	643 (92.8%)	466 (71.9%)		
Nodule diameter (mm)	5.4 (3.9)	6.3 (4.4)	4.4 (2.9)	9.264	<0.001
Nodule density (Hu)	56.7 (81.3)	54.3 (76.2)	61.1 (91.7)	0.291	0.773
Nodule distribution				31.391	<0.001
Multiple in both lungs	421 (31.4%)	256 (36.9%)	165 (25.5%)	20.483	<0.001
Multiple in the right lung	130 (9.7%)	70 (10.1%)	60 (9.3%)	0.271	0.603
Multiple in the left lung	50 (3.7%)	27 (3.9%)	23 (3.5%)	0.112	0.738
Single episode in the right superior lung	150 (11.2%)	76 (11.0%)	74 (11.4%)	0.069	0.793
Single episode in the right middle lung	163 (12.2%)	75 (10.8%)	88 (13.6%)	2.385	0.122
Single episode in the right inferior lung	186 (13.9%)	69 (10.0%)	117 (18.1%)	18.386	<0.001
Single episode in the left superior lung	101 (7.5%)	56 (8.1%)	45 (6.9%)	0.621	0.431
Single episode in the left inferior lung	140 (10.4%)	64 (9.2%)	76 (11.7%)	2.226	0.136
Accompanied radiological features	382 (28.5%)	159 (22.9%)	223 (34.4%)	198.576	0.012
Patchy shadow	164 (42.9%)	58 (36.5%)	106 (47.5%)	19.909	<0.001
Striped shadow	40 (10.5%)	5 (3.1%)	35 (15.7%)	25.342	<0.001
Mass shadow	27 (7.1%)	21 (13.2%)	6 (2.7%)	7.517	<0.001
Lymphadenectasis	40 (10.5%)	16 (10.1%)	24 (10.9%)	2.252	0.133
Bone destruction	18 (4.7%)	14 (8.9%)	4 (1.8%)	3.947	0.046
Pulmonary consolidation	20 (5.2%)	11 (6.9%)	9 (4.0%)	0.090	0.765
Emphysema	6 (1.6%)	4 (2.5%)	2 (0.9%)	0.107	0.744
Bronchiectasis	5 (1.3%)	2 (1.3%)	3 (1.3%)	0.006	0.940
Pulmonary cavity	11 (2.9%)	5 (3.1%)	6 (2.7%)	0.172	0.678
Internal lesions	11 (2.9%)	4 (2.5%)	7 (3.1%)	0.515	0.473
Pleural effusion	40 (10.4%)	19 (11.9%)	21 (9.4%)	0.288	0.591

### 3.2 General conditions and CT features between benign and malignant nodule subgroups

Based on the judgment of the radiologists and clinicians, a total of 209 cases with diagnosed nodules were detected. In the benign subgroup, 53.7% (n=51) were caused by infections, the rest were caused by thickened pleura, interstitial changes in both lungs, small contusions, hemangiomas, vascular malformations, etc. In the malignant nodule group, most of the nodules were metastases. 91% of the children showed a history of tumor. In terms of CT features, the nodule diameter (*P*<0.001), density (*P*=0.001), distribution (*P*<0.001) and accompanying radiological features (*P*=0.018) were markedly different between groups. Age (*P*=0.003) and pre-CT diagnosis (*P*<0.001) were statistically significant between the subgroups. No statistical difference showed in gender and clinical symptoms between subgroups (**Table 3**).

**Table 3:** Demographic and CT features of children with benign and malignant pulmonary nodules.

	All	Malignant	Benign	t/Z/x2	*P* value
	N=209	N=114	N=95		
Age (years)	6.9 (4.5)	6.1 (4.5)	7.9 (4.3)	3.017	0.003
Sex				1.566	0.211
Male	120 (57.4%)	61 (53.5%)	59 (62.1%)	1.566	0.211
Female	89 (42.6%)	53 (46.5%)	36 (37.9%)		
Respiratory symptoms				2.766	0.096
Yes	17 (8.1%)	6 (5.3%)	11 (11.6%)		
No	192 (91.9%)	108 (94.7%)	84 (88.4%)		
Nodule diameter (mm)	6.4 (4.5)	7.6 (5.2)	5.0 (3.1)	4.247	<0.001
Nodule density (Hu)	46.7 (24.4)	41.6 (18.4)	52.9 (29.1)	3.430	0.001
Nodule distribution				26.571	<0.001
Multiple in both lungs	73 (35.0%)	55 (48.2%)	18 (18.9%)	19.570	<0.001
Multiple in the right lung	21 (10.0%)	12 (10.5%)	9 (9.5%)	0.064	0.801
Multiple in the left lung	3 (1.4%)	1 (0.9%)	2 (2.1%)	0.025	0.873
Single episode in the right superior lung	27 (12.9%)	8 (7.0%)	19 (20.0%)	7.763	0.005
Single episode in the right middle lung	24 (11.5%)	13 (11.4%)	11 (11.6%)	0.002	0.968
Single episode in the right inferior lung	34 (16.3%)	12 (10.5%)	22 (23.2%)	6.070	0.014
Single episode in the left superior lung	8 (3.8%)	5 (4.5%)	3 (3.1%)	0.010	0.921
Single episode in the left inferior lung	19 (9.1%)	8 (7.0%)	11 (11.6%)	1.305	0.253
Accompanying radiological features	55 (26.3%)	21 (18.4%)	34 (35.8%)	39.438	0.018
Patchy shadow	13 (23.6%)	4 (19.0%)	9 (26.5%)	2.221	0.136
Striped shadow	4 (7.3%)	1 (4.8%)	3 (8.8%)	0.478	0.489
Mass shadow	5 (9.1%)	4 (19.0%)	1 (2.9%)	0.493	0.482
Lymphadenectasis	5 (9.1%)	3 (14.3%)	2 (5.9%)	0.000	1.000
Bone destruction	6 (10.9%)	3 (14.3%)	3 (8.8%)	0.000	1.000
Pulmonary consolidation	3 (5.5%)	0 (0.0%)	3 (8.8%)	-	0.092
Emphysema	1 (1.8%)	0 (0.0%)	1 (2.9%)	-	0.455
Bronchiectasis	2 (3.6%)	0 (0.0%)	2 (5.9%)	-	0.205
Pulmonary cavity	2 (3.6%)	0 (0.0%)	2 (5.9%)	-	0.205
Internal lesions	4 (7.3%)	0 (0.0%)	4 (11.8%)	-	0.041
Pleural effusion	10 (18.2%)	6 (28.6%)	4 (11.8%)	-	<0.001
Pre-CT diagnosis				73.686	<0.001
Tumor	159 (76.1%)	113 (99.1%)	46 (48.4%)	70.431	<0.001
Respiratory infections	9 (4.3%)	0 (0.0%)	9 (9.5%)	-	<0.001
Infection of other systems	1 (0.5%)	0 (0.0%)	1 (1.1%)	-	0.455
Immunodeficiencies	7 (3.3%)	0 (0.0%)	7 (7.4%)	-	0.004
Autoimmune diseases	11 (5.3%)	0 (0.0%)	11 (11.6%)	-	<0.001
Non-tumor hematological diseases	15 (7.2%)	0 (0.0%)	15 (15.7%)	-	<0.001
Others	7 (3.3%)	1 (0.9%)	6 (6.3%)	3.204	0.073

### 3.3 Decision tree model for determining the benignity or malignity of pediatric pulmonary nodules

**Figure 1:**
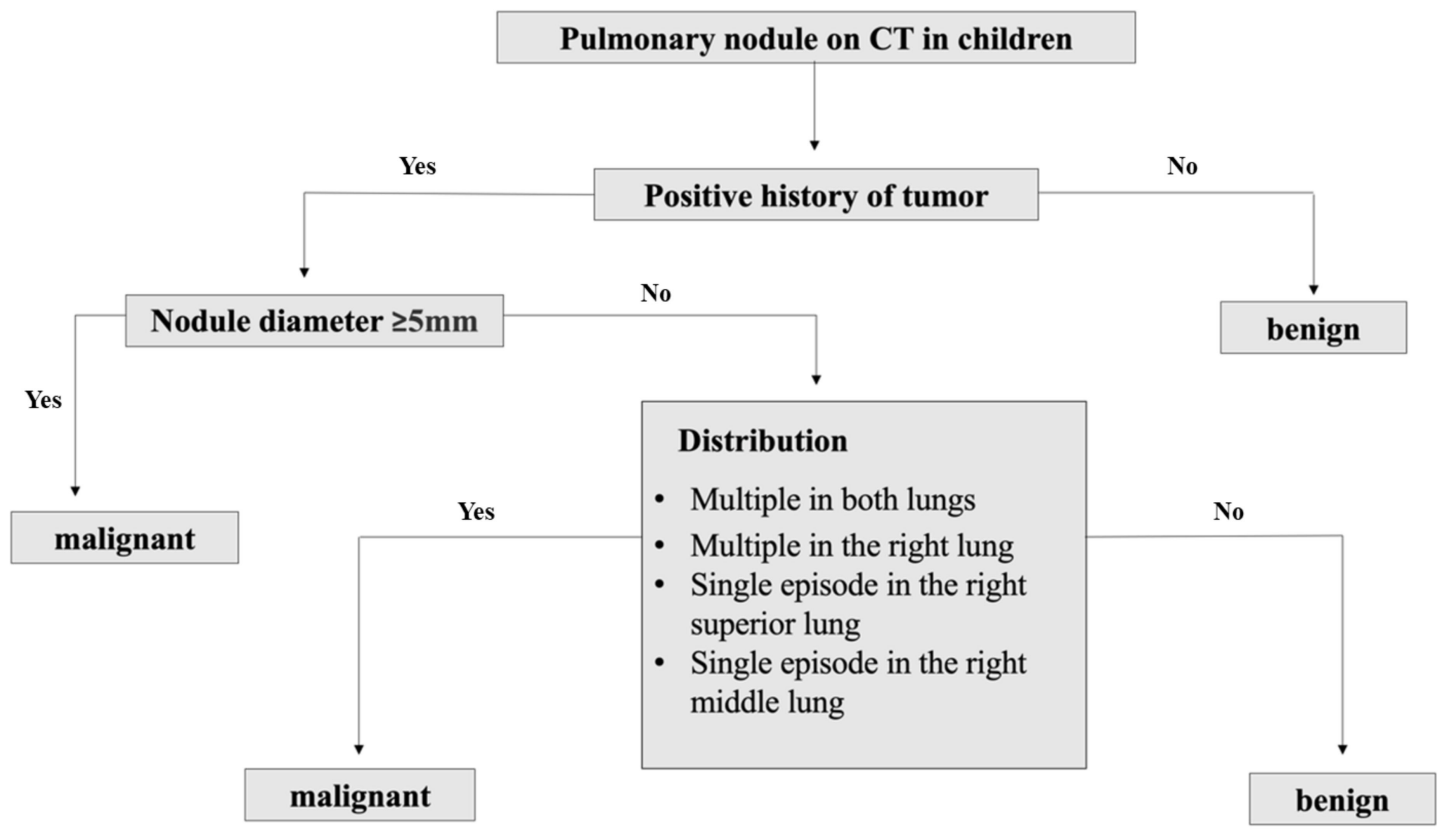
Clinical decision tree model of benign and malignant pulmonary nodules prediction in children.

Potential factors including age, gender, clinical symptoms, primary clinical diagnosis, nodule diameter, density, distribution location and accompanying radiological features were put into the decision tree model. After pruning strategies to achieve the best sensitivity and specificity, a clinical decision tree model was developed based on the primary clinical diagnosis, nodule diameter, and distribution pattern. This model has 3 specific nodes and 4 terminal nodes. Starting from the root node, it accurately distinguishes between the subgroups based on the threshold values of each classification or indicator. As the result showed in **Figure 1**, when the primary clinical diagnosis was not tumor, the nodule was considered benign; when the primary clinical diagnosis was tumor, further assessment of the nodule diameter was required. If the diameter was ≥5mm, the nodule was considered malignant, if it was <5mm, further assessment of the nodule distribution was necessary. Nodules multiple in both lungs, multiple only in the right lung, or solitary in the upper or middle right lobe are considered malignant, otherwise they were considered benign. Following the decision thresholds of the model, judgments are made step by step from the root node to determine the benignity versus malignity of pulmonary nodules.

**Figure 2:**
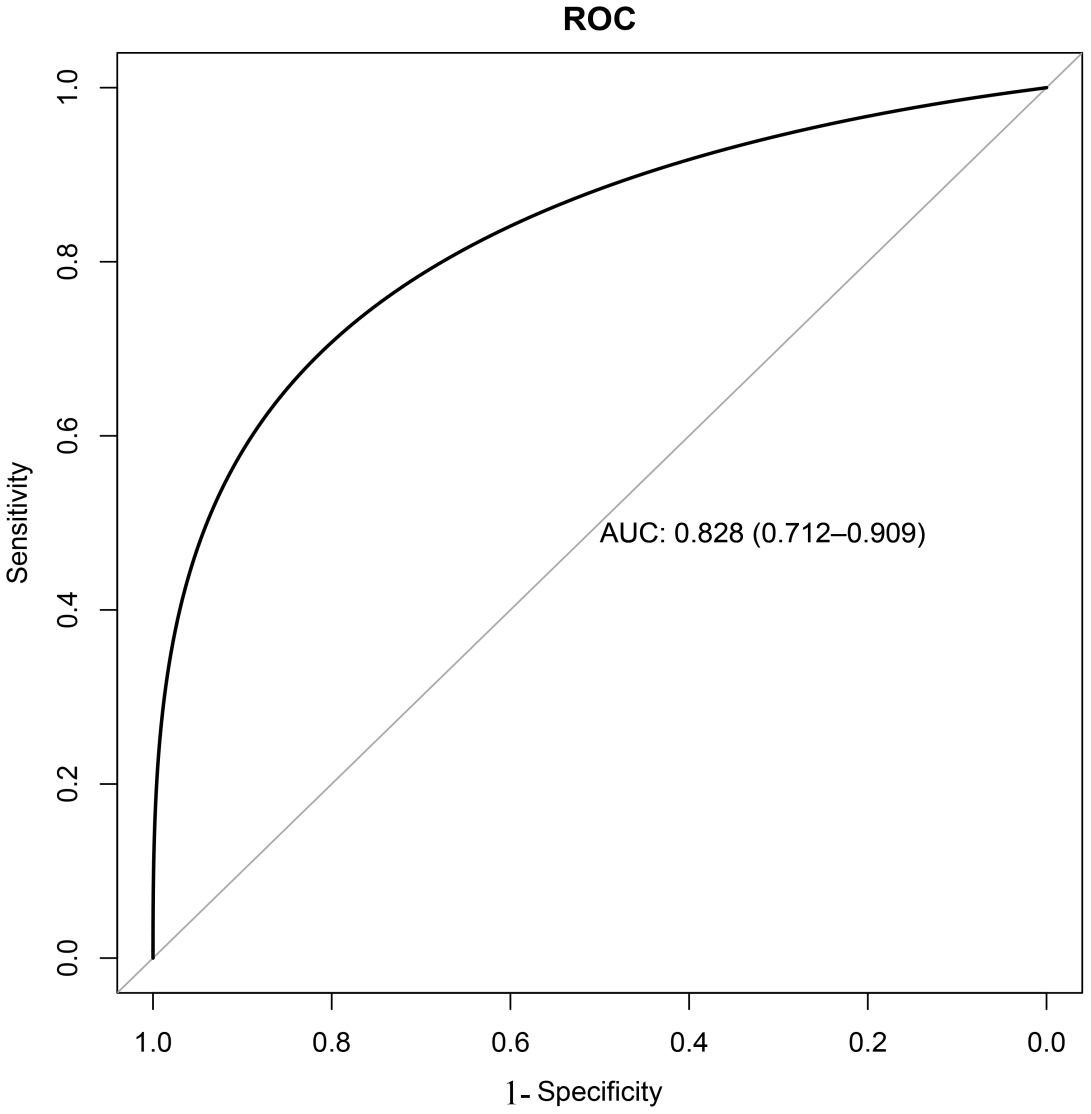
Receiver operating characteristic (ROC) curve of decision tree model validation.

Based on the training set, the results of the clinical decision tree for pediatric pulmonary nodules were compared with the original clinical diagnosis of the nodule nature. The optimal cutoff value was 34.00%. The sensitivity, specificity, accuracy and AUC in the validation set were 0.855, 0.833, 0.810 and 0.828 (95%CI: 0.712-0.909) respectively. The diagnostic model showed significantly high sensitivity (**Figure 2**).

## 4 Discussion

This study retrospectively analyzed the clinical and radiological characteristics of pulmonary nodules in 1341 children. The detection rate of pediatric pulmonary nodules was low. Children with the history of malignancies, nodule diameter ≥5mm, and specific nodule distribution (multiple in both lungs, multiple in the right lung, solitary in the upper or middle right lobe) were important predictable factors for malignity. Clinical and radiological dynamic follow-up was important for those patients.

The etiological spectrum of pulmonary nodules in children is different from that in adults. Clarifying the causes of pulmonary nodules is a challenge in their diagnosis and treatment. Previous studies have shown that many causes were associated with pediatric pulmonary nodules, such as infectious diseases, hematological disorders, autoimmune diseases, granulomatous diseases, obliterative bronchiolitis, pulmonary nodulosis, Crohn’s disease (17–19). In our finding, the primary clinical diagnoses before examination were malignant tumors, including hepatoblastoma, neuroblastoma, nephroblastoma, osteosarcoma, leukemia, lymphoma, which was consistent with previous study (3). We found The most common non-tumor cause was infection (including respiratory infections and other systemic infectious diseases). We also revealed that most pulmonary nodules in children are incidental. Only 17.3% of all children showed respiratory symptoms such as coughing, wheezing, shortness of breath or chest pain before CT scanning. In addition, children with a history of malignant tumors were younger, showed larger nodule diameters, and presented with multiple nodules often accompanied by osteolytic lesions and mass effect, which is consistent with previous findings (7, 8, 20).

The differentiation and etiological diagnosis of benign versus malignant pulmonary nodules is crucial in clinical management. Guidelines for adult pulmonary nodules offer detailed assessment and clinical management plans for physicians. Generally, nodules<6 mm are considered as low probability of malignancy (18), and nodules>5mm are recommended for clinical follow-up, those with solid nodules should undergo annual follow-up (21). Lung biopsy is the golden standard method for defining the nature of nodules and widely used in adult (22). One study (23) found that lung biopsy results led to changes in the management of 63% of children, particularly affecting the use of steroids. But biopsy is not frequently performed in children due to its invasiveness and parents’ worries. Therefore, the clinical features and radiological presentations of children are important clue to nodule assessment. In 2015, American Society for Pediatric Radiology’s Imaging Committee published the first guide for pediatric pulmonary nodules offering individualized management suggestions based on the CT characteristics and clinical history features (24, 25), but it lacks clear follow-up guidelines and criteria for judging benignity or malignancy. Several studies show that a history of malignant tumors, nodule diameter size, distribution, and margin can help in judging the nature of the nodule (8, 9, 15, 17, 26). Similarly, our decision tree model suggested that a nodule diameter≥5mm, a history of malignancies and nodule distribution are important predictors of malignancy. Multiple nodules also have predictable effect on malignancy (27). In our findings, not only multiple lesions (in the right lung or both lungs), solitary lesions in the upper or middle right lobe were also suggestive of malignancy.

Our study has some limitations. The selection bias exists due to the single-center study. Multicenter studies are highly recommended in the future with larger sample size to reduce bias. In addition, we failed to collect lung biopsy report of nodules in these cases, this reduced the reliability of the model. Biopsy is invasive with potential complications. Pathological evidence is hard to obtain when patient shows no indication for biopsy or surgery. We believe that further studies or meta-analysis are necessary to confirm our preliminary findings in the future.

In conclusions, our study demonstrated a history of malignancies, nodule diameter ≥5cm in size and distribution were potential predictors for malignant nodules. We suggested that children with potential malignant tumors have a higher probability of malignancy in pulmonary nodules. These patients require frequent follow-up and an early pulmonary nodule diagnosis.

## Data availability statement

The original contributions presented in the study are included in the article/supplementary material. Further inquiries can be directed to the corresponding authors.

## Ethics statement

The studies involving humans were approved by Institutional Review Board of the Shanghai Children’s Medical Center, Shanghai Jiao Tong University (SCMCIRB-K2022085-2). The studies were conducted in accordance with the local legislation and institutional requirements.

## Author contributions

MD: Conceptualization, Data curation, Investigation, Methodology, Validation, Writing – original draft, Writing – review & editing. SY: Data curation, Formal Analysis, Methodology, Writing – review & editing. HJ: Data curation, Supervision, Writing – original draft. YW: Data curation, Supervision, Writing – original draft. HM: Data curation, Supervision, Writing – original draft. SS: Data curation, Software, Supervision, Writing – original draft. JL: Conceptualization, Data curation, Formal Analysis, Methodology, Software, Supervision, Validation, Writing – review & editing. JC: Conceptualization, Methodology, Software, Supervision, Validation, Writing – review & editing. YY: Conceptualization, Data curation, Formal Analysis, Funding acquisition, Investigation, Methodology, Project administration, Resources, Software, Supervision, Validation, Visualization, Writing – review & editing, Writing – original draft.
